# Retraction notice for: “Risk factors for early-onset ventilator-associated pneumonia in aneurysmal subarachnoid hemorrhage patients” [Braz J Med Biol Res (2018) 51(7): e6830]

**DOI:** 10.1590/1414-431X20196830retraction

**Published:** 2020-01-13

J.B. Cui, Q.Q. Chen, T.T. Liu, S.J. Li

Neurosurgery Intensive Care Unit, Weifang People’s Hospital, Weifang, China

**Retraction for:** Braz J Med Biol Res | doi: http://10.1590/1414-431X20176830 | PMID: 29791584

The Brazilian Journal of Medical and Biological Research was contacted by one specialist questioning the validity of this study.

The Editors decided to retract the article: “Risk factors for early-onset ventilator-associated pneumonia in aneurysmal subarachnoid hemorrhage patients” that was published in volume 51 no. 7 (2018) (Epub May 17, 2018) in the Brazilian Journal of Medical and Biological Research <http://dx.doi.org/10.1590/1414-431X20176830> PMID: 29791584 | PMCID: PMC5972009

The authors agreed that this article should be retracted. All authors will be prohibited to publish in the Brazilian Journal of Medical and Biological Research in the future.

